# Improving the efficiency of ITO/nc-TiO_2_/CdS/P3HT:PCBM/PEDOT:PSS/Ag inverted solar cells by sensitizing TiO_2_ nanocrystalline film with chemical bath-deposited CdS quantum dots

**DOI:** 10.1186/1556-276X-8-453

**Published:** 2013-10-31

**Authors:** Chong Chen, Fumin Li

**Affiliations:** 1 Henan Key Laboratory of Photovoltaic Materials, Henan University, Kaifeng 475004, People’s Republic of China; 2 School of Physics and Electronics, Henan University, Kaifeng 475004, People’s Republic of China

**Keywords:** Inverted solar cell, Quantum dots, P3HT:PCBM, CdS, Efficiency

## Abstract

An improvement in the power conversion efficiency (PCE) of the inverted organic solar cell (ITO/nc-TiO_2_/P3HT:PCBM/PEDOT:PSS/Ag) is realized by depositing CdS quantum dots (QDs) on a nanocrystalline TiO_2_ (nc-TiO_2_) film as a light absorption material and an electron-selective material. The CdS QDs were deposited via a chemical bath deposition (CBD) method. Our results show that the best PCE of 3.37% for the ITO/nc-TiO_2_/CdS/P3HT:PCBM/PEDOT:PSS/Ag cell is about 1.13 times that (2.98%) of the cell without CdS QDs (i.e., ITO/nc-TiO_2_/P3HT:PCBM/PEDOT:PSS/Ag). The improved PCE can be mainly attributed to the increased light absorption and the reduced recombination of charge carriers from the TiO_2_ to the P3HT:PCBM film due to the introduced CdS QDs.

## Background

Organic bulk heterojunction (BHJ) photovoltaic (PV) cells have received considerable interest due to their advantages over their inorganic counterparts, such as low cost and large-area manufacture capability [1,2]. The organic PV cells have exhibited power conversion efficiencies of upward of 6% [3-6]. More recently, to improve the efficiency and the lifetime under outdoor conditions of the organic BHJ cell, the so-called inverted devices are reported. In inverted devices, metal oxides such as TiO_2_[7-13], ZnO [14-17], and Cs_2_CO_3_[18,19] are deposited on indium tin oxide (ITO) substrate and act as the electron-selective contact at the ITO interface. The solution composed of electron-donating and electron-accepting materials was then spin-coated on the metal oxide layer to form a photoactive layer. Then, the air-stable high-work-function metal (Ag) deposited on top of the active layer serves as the anodic electrode for hole collection.

It has been reported that ITO/nc-TiO_2_/P3HT:PCBM/Ag inverted solar cells under air mass 1.5 global (AM 1.5G) illumination have a low efficiency of 0.13% [11]. The main reason may be due to the low efficiency of charge collection at the interface between the active layer (P3HT:PCBM) and top metal electrodes. One of the main strategies usually employed to overcome this problem is to insert interfacial layer materials such as poly(3,4-ethylenedioxythiophene)/poly(styrenesulfonate) (PEDOT:PSS) [17], MoO_3_[19,20], WO_3_[11], and V_2_O_5_[21] between the active layer and anode (i.e., Ag electrode) to suppress the electron–hole recombination at the active layer/anode interface (i.e., P3HT:PCBM/Ag interface).

In this research, from another point of view, a new strategy is put forward to reduce the electron–hole recombination at the active layer/cathode interface (i.e., TiO_2_/P3HT:PCBM interface) by depositing CdS quantum dots (QDs) on a nanocrystalline TiO_2_ (nc-TiO_2_) film by chemical bath deposition (CBD) to enhance the efficiency of the ITO/nc-TiO_2_/P3HT:PCBM/PEDOT:PSS/Ag inverted solar cell without CdS QDs. The CBD method has been successfully used to deposit QDs onto the photoelectrodes to increase the light absorption in QD-sensitized solar cells [22]. However, this method is rarely used in organic BHJ PV cells. In this study, to improve the power conversion efficiency of the solar cells, the deposited CdS QDs on the nc-TiO_2_ film were used to increase the UV-visible (UV–vis) absorption of the cells and the interfacial area between the electron donor and electron acceptor. Moreover, CdS, an n-type semiconductor, can serve as an electron-selective layer to reduce the recombination between photogenerated electrons and holes.

**Figure 1:**
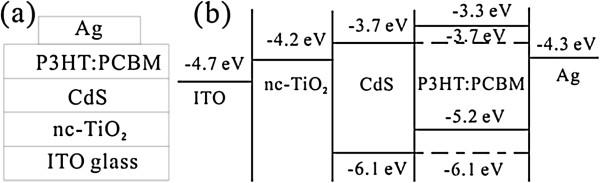
**Schematic diagram (a) and energy diagram (b) of the ITO/nc-TiO**_**2**_**/CdS/P3HT:PCBM/Ag device.**

In order to show more clearly the influence of CdS QDs on the performance of the ITO/nc-TiO_2_/CdS/P3HT:PCBM/Ag solar cell, the commonly inserted interfacial layer materials such as PEDOT:PSS between the P3HT:PCBM layer and Ag electrode are not used initially. The device architecture is shown schematically in Figure 1a, and the energy level diagrams of different materials used in the device fabrication are shown in Figure 1b. Then, to further improve the efficiency, the PEDOT:PSS as a hole-selective layer material is used in the ITO/nc-TiO_2_/CdS/P3HT:PCBM/PEDOT:PSS/Ag solar cell.

Our results show that the performance parameters, such as the short-circuit current density (*I*_sc_), the fill factor (FF), and the open-circuit photovoltage (*V*_oc_), of the cells with CdS increased largely compared to those of the cells without CdS QDs. As a result, the efficiency of ITO/nc-TiO_2_/CdS/P3HT:PCBM/PEDOT:PSS/Ag inverted solar cells increased to 3.37% from the efficiency of 2.98% of the ITO/nc-TiO_2_/P3HT:PCBM/Ag solar cell. This study reveals the importance of chemical bath-deposited CdS in enhancing the efficiency of ITO/nc-TiO_2_/P3HT:PCBM/Ag cells.

## Methods

### Formation of TiO_2_ nanocrystalline film on ITO substrate

The ITO-coated substrate is first cleaned by ultrasonic treatment in detergent and deionized (DI) water and then dried at 100°C for 10 min. The solution-processed nanocrystalline titania (TiO_2_) film was prepared as follows. A total of 0.2 g of titania nanoparticles (TiO_2_ P25, Degussa, Essen, Germany) was initially dissolved in a solution with 10 ml of ethanol and 10 ml of DI water, and then the TiO_2_ nanoparticle solution was stirred overnight. After that, the TiO_2_ solution was spin-coated onto the cleaned ITO substrate at 2,000 rpm, followed by baking on a hot plate at 150°C for 15 min to produce a TiO_2_ nanocrystalline film.

### Synthesis of ITO/nc-TiO_2_/CdS film

CdS nanoparticles were assembled on the ITO/nc-TiO_2_ film by CBD, as described elsewhere [22,23]. The prepared ITO/nc-TiO_2_ films were first dipped in a 0.1-M CdI_2_ aqueous solution for 10 s, in DI water for 10 s, in a 0.1-M Na_2_S solution for 10 s, and then in DI water for 10 s. Such an immersion procedure is considered one CBD cycle. In this study, the ITO/nc-TiO_2_ substrate after *n* cycles of CdS deposition was denoted as ITO/nc-TiO_2_/CdS(*n*) (*n* = 0, 5, 10, and 15). Note that for the ITO/nc-TiO_2_ substrate without CdS, *n* = 0.

### Preparation of ITO/nc-TiO_2_/CdS(*n*)/P3HT:PCBM/Ag and ITO/nc-TiO_2_/CdS(*n*)/P3HT:PCBM/PEDOT:PSS/Ag solar cells

After transferring the substrates into a N_2_ glove box, the poly(3-hexylthiophene) (P3HT; Rieke Metals, Lincoln, NE, USA)/[6]-phenyl-C61-butyric acid methyl ester (PCBM; Nano-C, Westwood, MA, USA) (P3HT:PCBM) blend film was deposited onto an ITO/nc-TiO_2_ ITO/nc-TiO_2_/CdS(*n*) film by spin coating a 1,2-dichlorobenzene (DCB) solution that contains P3HT (20 mg/ml) and PCBM (20 mg/ml) with a weight ratio of 1:1 at 400 rpm for 90 s in a N_2_-filled glove box, resulting in an active layer of about 250 nm. Then, the ITO/nc-TiO_2_/CdS(*n*)/P3HT:PCBM films were thermally annealed on a hot plate at 150°C for 15 min (*n* = 0, 5, 10, and 15). Finally, the silver electrode (*ca.* 80 nm) was thermally evaporated at low pressure (<1 × 10^−6^ Torr). The active area of the device was about 0.04 cm^2^. For the ITO/nc-TiO_2_/CdS(*n*)/P3HT:PCBM/PEDOT:PSS/Ag devices (*n* = 0, 5, 10, and 15), the hole-selective layer of PEDOT:PSS (Clevios P VP Al 4083, Leverkusen, Germany) was spin-coated onto the prepared ITO/nc-TiO_2_/CdS(*n*)/P3HT:PCBM films from its isopropanol solution at 4,000 rpm for 1 min. After that, the films were baked at 150°C for 10 min. Finally, the silver electrode was thermally evaporated. For each type of solar cells, 12 devices are fabricated to compare the performance of the cells.

### Characterization and measurements

UV–vis diffuse reflectance spectroscopy (DRS) was carried out using an S-4100 spectrometer with a SA-13.1 diffuse reflector (Scinco Co. LTD, Seoul, South Korea). The optical absorption of the P3HT/PCBM blend films was measured using a PerkinElmer 35 UV-visible spectrophotometer on quartz substrates (Waltham, MA, USA). The atomic force microscopy (AFM) measurements were performed using an Agilent 5500 AFM (Agilent Technologies, Chandler, AZ, USA). Field emission transmission electron microscopy (FETEM; Model Fei Nova 230, FEI Company, Hillsboro, OR, USA) measurements were carried out by scratching a portion of the CdS/TiO_2_ sample, followed by ultrasonication for a few minutes. Then, a drop of ethanol was placed on a copper grid and subjected to high-resolution transmission electron microscopy (HRTEM). Transmission electron microscopy (TEM) analyses were carried out on a Tecnai G2 F30 TEM (FEI Company, Hillsboro, OR, USA). The crystalline phase and structure of the as-prepared ITO/nc-TiO_2_/CdS film were confirmed by power X-ray diffractometry (XRD; DX-2500; Dandong Fangyuan Instrument Co., Ltd., Dandong, China). Current density-voltage (*I*-*V*) characteristics of the as-prepared devices were measured using a Keithley 2410 source meter (Cleveland, OH, USA) in the dark and under the illumination of AM 1.5G simulated solar light (100 mW/cm^2^) provided by a solar simulator (Newport Inc., Irvine, CA, USA).

## Results and discussion

**Figure 2:**
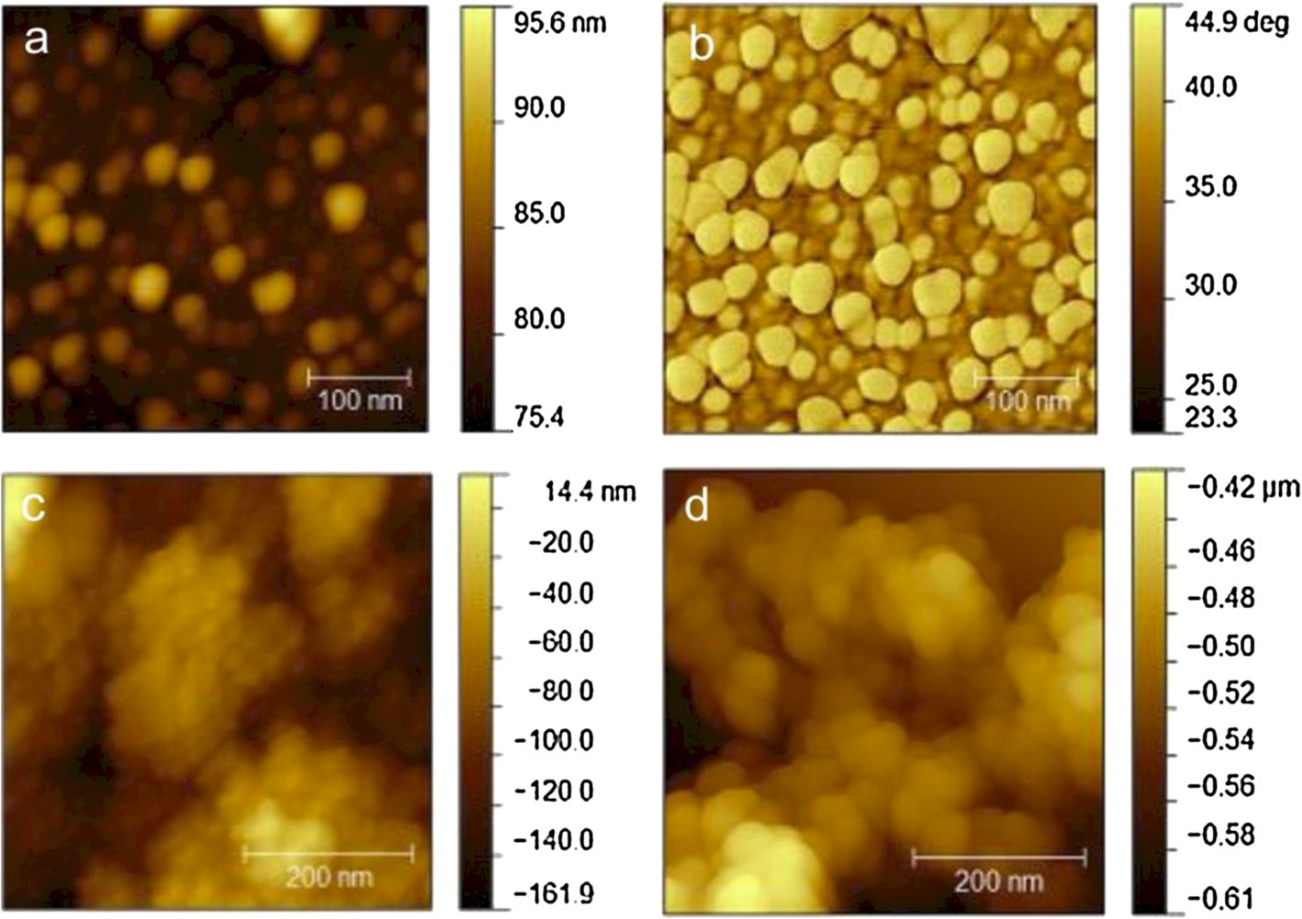
**AFM images of the films. (a)** The AFM topography image and **(b)** the corresponding AFM phase image of the ITO/nc-TiO_2_ film. The AFM topography images of **(c)** the ITO/nc-TiO_2_/CdS(5) film and **(d)** the ITO/nc-TiO_2_/CdS(15) film.

Figure 2a shows the AFM topography image of the ITO/nc-TiO_2_ thin film. To show the nc-TiO_2_ film on the ITO glass substrate more clearly, the corresponding AFM phase image of the ITO/nc-TiO_2_ thin film is shown in Figure 2b. It can be seen that the TiO_2_ nanoparticles are distributed uniformly on the ITO glass, and the size of single particle is between 20 nm and 50 nm, which is consistent with the average size (25 nm) of P25 TiO_2_ nanoparticles. The root-mean-square (rms) surface roughness value of the ITO/nc-TiO_2_ for 0.5 × 0.5 μm^2^ is about 12 nm (Figure 2a).

Figure 2c shows the AFM topography image of the ITO/nc-TiO_2_/CdS(5) thin film. The CdS nanoparticles can be clearly found in Figure 2c, and the dense CdS nanocrystalline film has been formed. The roughness of the ITO/nc-TiO_2_/CdS(5) thin film for 0.5 × 0.5 μm^2^ is about 48 nm, which is much higher than that of the TiO_2_ nanocrystalline film, suggesting that the introduction of CdS nanoparticles may lead to a more larger interfacial area between the electron donor and acceptor. In our case, the increased roughness of the ITO/nc-TiO_2_/CdS/P3HT:PCBM film may provide an increased interface area between the P3HT and TiO_2_ or CdS compared to the ITO/nc-TiO_2_/P3HT:PCBM film without CdS, which obviously would increase the interfacial dissociation probability of photogenerated excitons at the P3HT/CdS and P3HT/TiO_2_ interfaces and thereby increase the photocurrent density of the cells [24]. For a comparison, the AFM topography image of the ITO/nc-TiO_2_/CdS(15) thin film is also shown in Figure 2d. After the introduction of 15 cycles of CdS deposition, the size of the CdS nanoparticle increased slightly. Importantly, the roughness is about 80 nm, which is higher than that of the ITO/nc-TiO_2_/CdS(5) film, suggesting that the roughness of the ITO/nc-TiO_2_/CdS thin film increases with the cycle number of CdS deposition.

**Figure 3:**
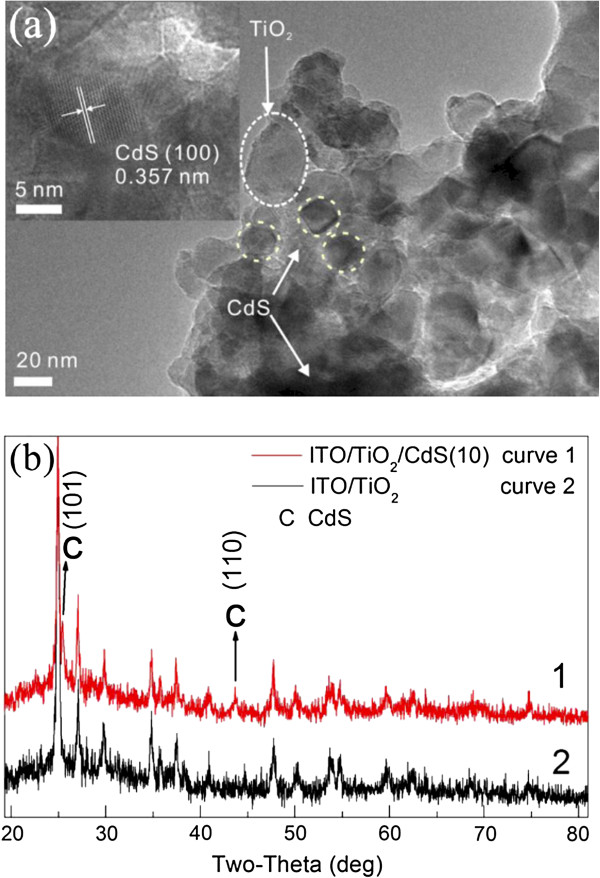
**TEM images and XRD patterns of the films. (a)** TEM images of the ITO/nc-TiO_2_/CdS(5) film at low and high (inset) magnifications. **(b)** XRD patterns of the as-prepared ITO/nc-TiO_2_ and ITO/nc-TiO_2_/CdS(10) films. C represents CdS. The large particles are titania Degussa P25 nanoparticles. The small dark spots belong to CdS nanoparticles with diameters of about 10 to15 nm.

TEM was carried out to characterize the detailed microscopic structure of the ITO/nc-TiO_2_/CdS(5) film. Figure 3a shows the low-resolution TEM image of the ITO/nc-TiO_2_/CdS(5) film. It can be found that CdS nanoparticles with average diameters of about 10 nm can be distinguished as dark spots, in which TiO_2_ P25 nanoparticles with average diameters of about 25 nm can be distinguished as bright spots. The inset of Figure 3a shows the high-resolution (HR) TEM image of TiO_2_/CdS(5), in which the lattice spacing of 0.357 nm is assigned to the (100) plane of the hexagonal phase of CdS (JCPDS 80–0006), which is in good agreement with our previous report [22].

Figure 3b shows the XRD patterns of the as-prepared ITO/nc-TiO_2_/CdS(10) (curve 1) and ITO/nc-TiO_2_ (curve 2) films. By carefully comparing the diffraction peaks in curves 1 and 2, it can be found that the intensities of two peaks at 2*θ* = 28.3° and 43.9° (corresponding to the (101) and (110) faces of CdS, respectively) in the ITO/nc-TiO_2_/CdS(10) film are greater than the intensities of those in the plain ITO/nc-TiO_2_ film, indicating the formation of the hexagonal-phase CdS.

**Figure 4:**
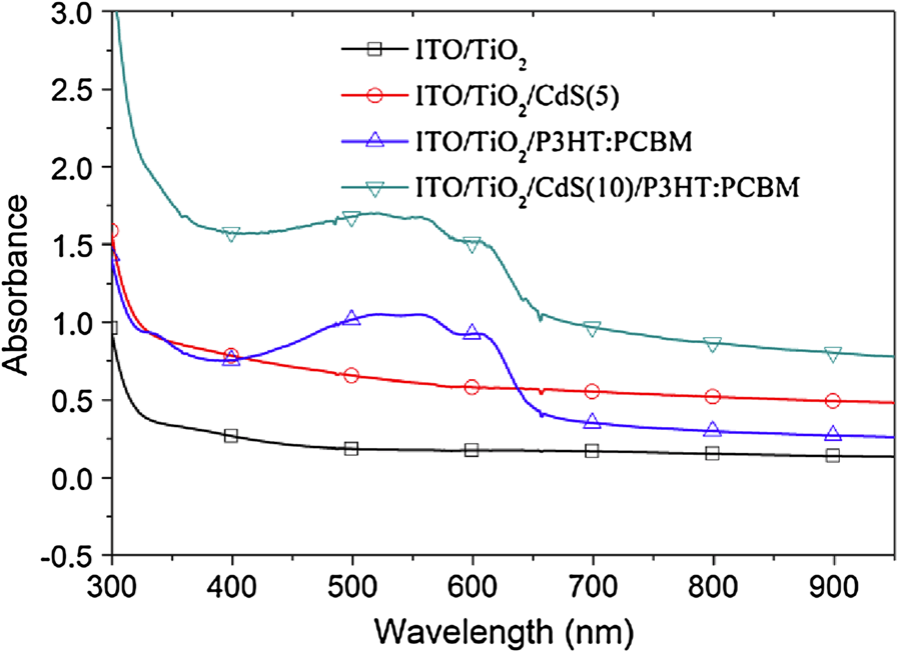
**UV–vis absorption spectrum of the ITO/nc-TiO**_**2**_**, ITO/nc-TiO**_**2**_**/CdS(5), and ITO/nc-TiO**_**2**_**/CdS(*n*)/P3HT:PCBM films (*n*** **= 0 and 10).**

To investigate the influence of CdS on the optical properties of the ITO/nc-TiO_2_ and ITO/nc-TiO_2_/P3HT:PCBM films, the UV–vis absorption spectra of the ITO/nc-TiO_2_, ITO/nc-TiO_2_/CdS(5), ITO/nc-TiO_2_/P3HT:PCBM, and ITO/nc-TiO_2_/CdS(10)/P3HT:PCBM films are shown in Figure 4. It can be seen that compared to that of the ITO/nc-TiO_2_ film without CdS, the absorbance of the spectra of the ITO/nc-TiO_2_/CdS(5) film increases largely in the 300- to 950-nm wavelength region, which is similar to that for the CdS nanoparticle-coated TiO_2_ nanotube film [22,23]. Apparently, the deposited CdS nanoparticles contribute to the spectral response. Similarly, compared to that of the ITO/nc-TiO_2_/P3HT:PCBM film, after the introduction of CdS deposition, the light absorption of the ITO/nc-TiO_2_/CdS(10)/P3HT:PCBM film in the measured wavelength region increased, which is similar to that of CdS/P3HT composite layers [25]. It is known that the optical properties of CdS QD-sensitized TiO_2_ are directly affected by the size of the CdS QDs due to the quantum size effect [26-28]. In our case, increasing the number of CdS deposition cycles leads to a progressive aggregation of the CdS QDs, which results in a concomitant redshift of the absorption feature. However, the quantum size effect cannot be used to explain the increased light absorption of the ITO/nc-TiO_2_/CdS(5) and ITO/nc-TiO_2_/CdS(10)/P3HT:PCBM films in near-infrared (NIR) region (wavelength >700 nm) because bulk CdS with an absorption onset of 2.42 eV mainly absorbs in the visible region (wavelength from roughly 400 to 700 nm). The increased light absorption of these films with CdS in the NIR region may be probably due to the electron coupling between the TiO_2_ and CdS heterostructure [29,30]. As shown in Figure 1b, the photogenerated electrons can effectively transfer from the conduction band (CB) of CdS to that of TiO_2_ because of the lower CB level (−4.2 eV) of TiO_2_ than that (−3.7 eV) of CdS, which may most probably be due to a superposition of the electronic states of TiO_2_ and CdS. Therefore, an electronic interaction between the TiO_2_ and CdS exists and makes the bandgap of the TiO_2_/CdS composite system different from that of TiO_2_ or CdS. For example, as reported previously by Luo et al. [30], the bandgap of the TiO_2_/CdS composite system is 2.39 eV, which is even smaller than that of bulk CdS and leads to a weak absorption of the TiO_2_/CdS film in the NIR region. These results show that the deposited CdS nanoparticles effectively improve the light absorption of the ITO/nc-TiO_2_ and ITO/nc-TiO_2_/P3HT:PCBM films, which is beneficial to the improvement of the performance of the cells.

**Figure 5:**
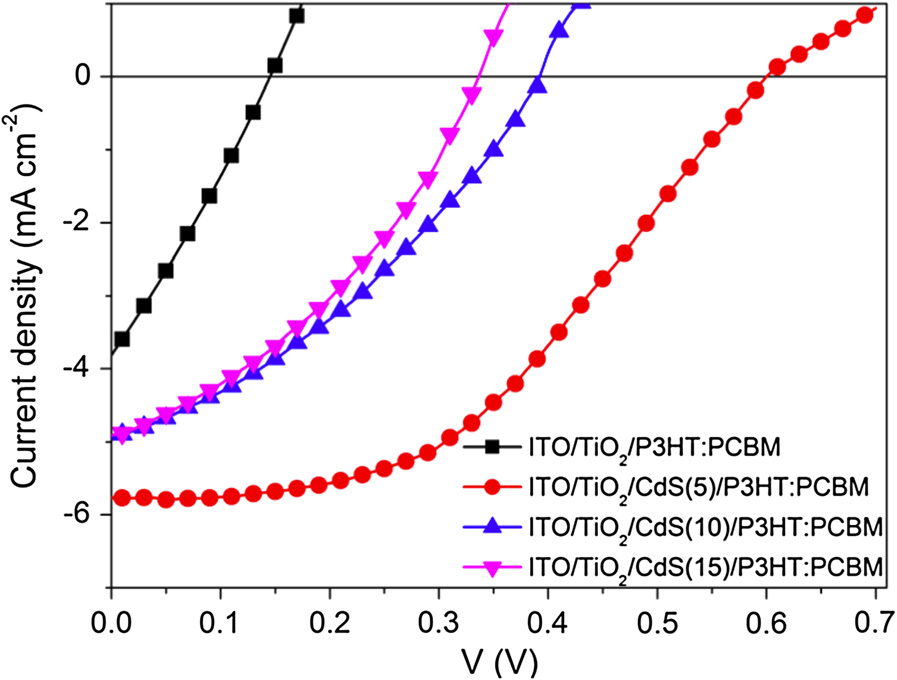
***I*-*V *characteristics of the ITO/nc-TiO**_**2**_**/CdS( *n *)/P3HT:PCBM devices (*n*** **= 0, 5, 10, and 15).**

In order to more clearly investigate the influence of CdS QDs on the optoelectronic performance of the prepared solar cells, the *I*-*V* characteristics of the ITO/nc-TiO_2_/CdS(*n*)/P3HT:PCBM solar cells without the PEDOT:PSS layer under 100-mW/cm^2^ white light illumination were first measured as shown in Figure 5 (*n* = 0, 5, 10, and 15). Four factors concerning cell performance: *V*_oc_, *I*_sc_, fill factor (FF), and power conversion efficiency (PCE), extracted from the *I*-*V* characteristics are shown in Table 1. It can be found that the PCE of the ITO/nc-TiO_2_/P3HT:PCBM/Ag cell under white light illumination with an intensity of 100 mW/cm^2^ is only about 0.15%, which is comparable to the reported PCE value of 0.13% [11]. Moreover, the *V*_oc_ (0.15 V), *I*_sc_ (3.81 mA/cm^2^), and FF (0.27) are also very close to the reported values, i.e., *V*_oc_ = 0.15 V, *I*_sc_ = 4 mA/cm^2^, and FF = 0.27 [11].

**Table 1:** **Summary of PV cell performance under white light illumination with an intensity of 100 mW/cm**^**2**^

**Cells**	***V***_**oc **_**(V)**	***I***_**sc **_**(mA/cm**^**2**^**)**	**PCE (%)**	**FF**
ITO/nc-TiO_2_/P3HT:PCBM/Ag	0.15	3.81	0.15	0.27
ITO/nc-TiO_2_/CdS(5)/P3HT:PCBM/Ag	0.60	5.81	1.57	0.5
ITO/nc-TiO_2_/CdS(10)/P3HT:PCBM/Ag	0.40	4.93	0.68	0.35
ITO/nc-TiO_2_/CdS(15)/P3HT:PCBM/Ag	0.33	4.90	0.61	0.36

After the introduction of CdS, all the performance parameters of ITO/nc-TiO_2_/CdS(*n*)/P3HT:PCBM/Ag cells: *V*_oc_, *I*_sc_, PCE, and FF, are significantly improved compared to the cells without CdS, i.e., ITO/nc-TiO_2_/P3HT:PCBM/Ag cell. After five cycles of CdS deposition, the cell of ITO/nc-TiO_2_/CdS(*n*)/P3HT:PCBM/Ag gives rise to a significant increase in *V*_oc_, which increases from 0.15 to 0.60, 0.40, and 0.33 V for *n* = 5, 10, and 15, respectively. This result can be explained as follows. On one hand, it is known that *V*_oc_ is mainly dominated by the energy level difference between the donor highest occupied molecular orbital (HOMO) and the acceptor lowest unoccupied molecular orbital (LUMO) levels in the polymer bulk heterojunction solar cells. In our case, before the deposition of CdS, the electron acceptor materials are TiO_2_ and PCBM. However, after the introduction of CdS, CdS also works as an electron acceptor. Apparently, the effective LUMO level of the acceptor should be determined by three acceptor materials, i.e., TiO_2_, PCBM, and CdS. Importantly, the CB level (−3.7 eV) of CdS is higher than that (−4.2 eV) of TiO_2_[22], which probably enhances the effective LUMO level of the acceptor and the energy level difference between the HOMO of donor and the LUMO of acceptor levels, ultimately increasing the *V*_oc_ of the cells with CdS compared to the ITO/nc-TiO_2_/P3HT:PCBM/Ag cell without CdS. On the other hand, *V*_oc_ may also be affected by charge recombination in the cells under open-circuit condition. CdS as an electron-selective layer can prevent the electron from escaping the TiO_2_ to the active layer, which can be characterized by the shunt resistance (*R*_sh_), calculated from the inverse slope of *I*-*V* characteristics under illumination at *V* = 0 V. A higher *R*_sh_ is more beneficial to the increase of *V*_oc_. This explanation is supported by the shunt resistance of the ITO/nc-TiO_2_/CdS(*n*)/P3HT:PCBM/Ag cells: 620, 350, and 290 Ω/cm^2^, for *n* = 5, 10, and 15, respectively, indicating an increased shunt resistance compared to the ITO/nc-TiO_2_/P3HT:PCBM/Ag without CdS.

Besides, the improvement in both *I*_sc_ and FF of the ITO/nc-TiO_2_/CdS(*n*)/P3HT:PCBM/Ag cells is also found. There are several reasons for *I*_sc_ enhancement. The first one may be the reduced charge recombination from TiO_2_ to the P3HT:PCBM film when introducing CdS nanoparticles. It can be seen from the energy diagram shown in Figure 1b that the photogenerated electrons are injected from CdS and P3HT to TiO_2_ and PCBM, part of which may combine with the holes in P3HT. However, compared to the cells without CdS, the recombination in the cells with CdS is reduced because of the formation of the CdS energy barrier layer, which is similar to the case of CdS-sensitized TiO_2_ nanotube arrays [22]. The increased interfacial area between the donor and acceptor as shown in Figure 2 after the deposition of CdS on TiO_2_ may be the second reason, which makes more excitons dissociate into free electrons and holes. The third reason may be the enhanced UV–vis absorption after the deposition of CdS (see Figure 4). The more absorbed light will lead to more charges and therefore increasing the *I*_sc_.

The reason for the increase in FF can be attributed to the increased *R*_sh_ as discussed above compared to the cells without CdS. For the ITO/nc-TiO_2_/CdS(*n*)/P3HT:PCBM/Ag cells, however, with the increase of CdS cycle number *n* from 5 to 15, the *V*_oc_ decreased from 0.6 to 0.33 V. The *I*_sc_ decreased from 5.81 to 4.9 mA/cm^2^ and the FF decreased from 0.50 to about 0.36. These results might be caused by the increased roughness of the ITO/nc-TiO_2_/CdS(*n*)/P3HT:PCBM/Ag cells with the increase in cycle number *n*. On one hand, the CdS nanocrystalline film can prevent the charge transfer back from TiO_2_ to the P3HT:PCBM film. On the other hand, the increased absorption amount of CdS will increase the roughness of the ITO/nc-TiO_2_/CdS films as shown in Figure 2, which might lead to form small CdS nanoparticle islands instead of a uniform film. Some of these islands may not be fully covered by the P3HT:PCBM film, which leads to increased leakage current in the cells and therefore decreasing the *V*_oc_ and *I*_sc_. The decrease in FF may be due to the reduced *R*_sh_, which decreased from about 67 to about 21 Ω/cm^2^ with the increase of *n* from 5 to 10 (Figure 5). Finally, the PCE of the ITO/nc-TiO_2_/CdS(*n*)/P3HT:PCBM/Ag cells decreased from 1.57% to 0.61% (Table 1), which is still higher than that (0.15%) of the ITO/nc-TiO_2_/P3HT:PCBM/Ag cell. Nonetheless, our results clearly show that the PCE of the ITO/nc-TiO_2_/CdS(*n*)/P3HT:PCBM/Ag cells increased significantly by depositing CdS on TiO_2_. The best PCE of 1.57% for the ITO/nc-TiO_2_/CdS(5)/P3HT:PCBM/Ag cell is achieved, which is about ten times that (0.15%) of the ITO/nc-TiO_2_/P3HT:PCBM/Ag cell. To sum up, the three main reasons for the improved efficiency of the ITO/nc-TiO_2_/CdS/P3HT:PCBM/Ag cells are as follows: first, the absorbance of the spectra of the ITO/nc-TiO_2_/CdS/P3HT:PCBM film increased significantly due to the deposited CdS QDs; second, the deposited CdS layer between the nc-TiO_2_ and active layer (P3HT:PCBM) can reduce the charge recombination as an energy barrier layer; and third, the interfacial area increased due to the increased roughness of the ITO/nc-TiO_2_/CdS film compared to the ITO/nc-TiO_2_ without CdS QDs, which makes more excitons dissociate into free electrons and holes at the P3HT/CdS and P3HT/TiO_2_ interfaces.

**Figure 6:**
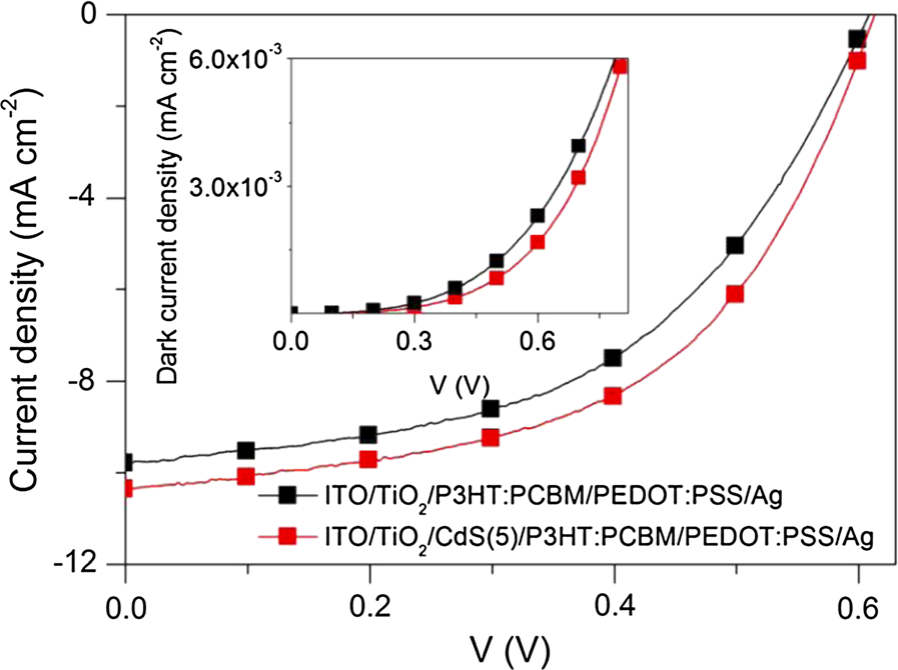
***I*-*V *characteristics of the ITO/nc-TiO**_**2**_**/P3HT:PCBM/PEDOT:PSS/Ag and ITO/nc-TiO**_**2**_**/CdS(5)/P3HT:PCBM/PEDOT:PSS/Ag solar cells.** Under an AM 1.5G (100 mW/cm^2^) condition and in the dark (inset).

According to the above results, it should be expected that the efficiency of the ITO/nc-TiO_2_/CdS/P3HT:PCBM/Ag cell can be further improved by inserting interfacial layer materials such as PEDOT:PSS between the P3HT/PCBM layer and the anode (Ag). As an example, the *I*-*V* characteristics of the best ITO/nc-TiO_2_/P3HT:PCBM/PEDOT:PSS/Ag and ITO/nc-TiO_2_/CdS(5)/P3HT:PCBM/PEDOT:PSS/Ag devices under an AM 1.5G (100 mW/cm^2^) condition and in the dark are shown in Figure 6. It can be seen that compared to that of the ITO/nc-TiO_2_/P3HT:PCBM/PEDOT:PSS/Ag, the *V*_oc_ of the ITO/nc-TiO_2_/CdS(5)/P3HT:PCBM/PEDOT:PSS/Ag only increased slightly; however, the *I*_sc_ increased from 9.8 to 10.3 mA/cm^2^ and the FF increased from 0.52 to 0.55. As a result, the efficiency of 3.37% achieved by the ITO/nc-TiO_2_/CdS(5)/P3HT:PCBM/PEDOT:PSS/Ag is about 13% higher than that (2.98%) of the ITO/nc-TiO_2_/P3HT:PCBM/PEDOT:PSS/Ag without CdS. As discussed above, one of the reasons for the improved efficiency of the ITO/nc-TiO_2_/CdS(*n*)/P3HT:PCBM/PEDOT:PSS/Ag cells with CdS is reduced charge recombination in the cells due to the formation of CdS on the nc-TiO_2_ layer as an energy barrier layer. The charge recombination in organic solar cells can be represented by the dark current [31,32]. To support this explanation, the *I*-*V* characteristics of the best ITO/nc-TiO_2_/P3HT:PCBM/PEDOT:PSS/Ag and ITO/nc-TiO_2_/CdS(5)/P3HT:PCBM/PEDOT:PSS/Ag devices in the dark are shown in the inset of Figure 6. It can be found that the dark current density of the ITO/nc-TiO_2_/CdS(5)/P3HT:PCBM/PEDOT:PSS/Ag device is much smaller than that of the ITO/nc-TiO_2_/P3HT:PCBM/PEDOT:PSS/Ag device without CdS, which indicates that the charge recombination is suppressed by the deposited CdS nanoparticles. This result further confirmed the effectiveness of the chemical bath-deposited CdS on the nc-TiO_2_ film that can effectively reduce the charge recombination and improve the power conversion efficiency of the inverted polymer solar cells.

## Conclusions

CdS nanoparticles were deposited on a nc-TiO_2_ film by chemical bath deposition to improve the power conversion efficiency of the inverted solar cell with a device structure of ITO/nc-TiO_2_/P3HT:PCBM/PEDOT:PSS/Ag. In the case of ITO/nc-TiO_2_/CdS/P3HT:PCBM/PEDOT:PSS/Ag, deposited CdS does not only enhance the optical absorption but also suppresses the charge recombination. Finally, compared to that (2.98%) of the ITO/nc-TiO_2_/P3HT:PCBM/PEDOT:PSS/Ag, the power conversion efficiency of the ITO/nc-TiO_2_/CdS/P3HT:PCBM/PEDOT:PSS/Ag cell under white light illumination with an intensity of 100 mW/cm^2^ increased to 3.37% due to the increased optical absorption and the reduced recombination.

## Competing interests

The authors declare that they have no competing interests.

## Authors’ contributions

CC carried out the experiments, participated in the sequence alignment, and drafted the manuscript. FL participated in the device preparation. Both authors read and approved the final manuscript.
